# Autoencoder-Assisted Stacked Ensemble Learning for Lymphoma Subtype Classification: A Hybrid Deep Learning and Machine Learning Approach

**DOI:** 10.3390/tomography11080091

**Published:** 2025-08-18

**Authors:** Roseline Oluwaseun Ogundokun, Pius Adewale Owolawi, Chunling Tu, Etienne van Wyk

**Affiliations:** Department of Computer Systems Engineering, Tshwane University of Technology (TUT), Pretoria 0001, South Africa; owolawipa@tut.ac.za (P.A.O.); duc@tut.ac.za (C.T.); vanwykea@tut.ac.za (E.v.W.)

**Keywords:** lymphoma classification, autoencoder, stacked ensemble learning, deep feature extraction, digital pathology, machine learning

## Abstract

Background: Accurate subtype identification of lymphoma cancer is crucial for effective diagnosis and treatment planning. Although standard deep learning algorithms have demonstrated robustness, they are still prone to overfitting and limited generalization, necessitating more reliable and robust methods. Objectives: This study presents an autoencoder-augmented stacked ensemble learning (SEL) framework integrating deep feature extraction (DFE) and ensembles of machine learning classifiers to improve lymphoma subtype identification. Methods: Convolutional autoencoder (CAE) was utilized to obtain high-level feature representations of histopathological images, followed by dimensionality reduction via Principal Component Analysis (PCA). Various models were utilized for classifying extracted features, i.e., Random Forest (RF), Support Vector Machine (SVM), Multi-Layer Perceptron (MLP), AdaBoost, and Extra Trees classifiers. A Gradient Boosting Machine (GBM) meta-classifier was utilized in an SEL approach to further fine-tune final predictions. Results: All the models were tested using accuracy, area under the curve (AUC), and Average Precision (AP) metrics. The stacked ensemble classifier performed better than all the individual models with a 99.04% accuracy, 0.9998 AUC, and 0.9996 AP, far exceeding what regular deep learning (DL) methods would achieve. Of standalone classifiers, MLP (97.71% accuracy, 0.9986 AUC, 0.9973 AP) and Random Forest (96.71% accuracy, 0.9977 AUC, 0.9953 AP) provided the best prediction performance, while AdaBoost was the poorest performer (68.25% accuracy, 0.8194 AUC, 0.6424 AP). PCA and t-SNE plots confirmed that DFE effectively enhances class discrimination. Conclusion: This study demonstrates a highly accurate and reliable approach to lymphoma classification by using autoencoder-assisted ensemble learning, reducing the misclassification rate and significantly enhancing the accuracy of diagnosis. AI-based models are designed to assist pathologists by providing interpretable outputs such as class probabilities and visualizations (e.g., Grad-CAM), enabling them to understand and validate predictions in the diagnostic workflow. Future studies should enhance computational efficacy and conduct multi-centre validation studies to confirm the model’s generalizability on extensive collections of histopathological datasets.

## 1. Introduction

Machine learning (ML), especially DL, has revolutionized medical image analysis recently [1,2]. Deep neural networks (DNNs) can automatically learn hierarchical features from imaging data, enabling state-of-the-art performance on disease detection and classification tasks [3,4]. DL models often outperform traditional ML methods that rely on hand-crafted features and, in some cases, even surpass the diagnostic accuracy of human experts like radiologists [5,6]. For example, a study comparing convolutional neural networks (CNNs) with conventional classifiers for detecting pathology in CT scans found that CNNs slightly outperformed the best traditional model (area under the curve 0.982 vs. 0.975) [7]. These successes have fueled high expectations that artificial intelligence (AI) will bring transformative improvements to healthcare [8,9]. At the same time, integrating AI into clinical practice faces challenges—deep learning models are “data-hungry” and require large, labelled datasets, often scarce in medicine [10,11]. This has motivated research into methods that can better use limited data, such as unsupervised representation learning and model ensemble techniques. One domain of particular interest is digital pathology, where whole-slide images are analyzed to aid disease diagnosis.

Lymphomas (lymphatic system cancers) present a diagnostic challenge: subtyping them requires expert knowledge, extensive experience, and ancillary tests like immunohistochemistry and molecular analyses [10,12]. These requirements are not always available, and the number of specialized pathologists is declining, making automated decision support highly desirable [13,14]. Digital pathology tools leveraging AI have already shown promise in classifying diseases—for instance, AI methods have successfully differentiated histopathology subtypes of carcinomas [13,14,15]. In the context of lymphoma, deep learning approaches have begun to emerge. Litjens et al. [1] highlighted deep learning as a “methodology of choice” in medical imaging and noted its rapid proliferation across pathology and other specialities [1,16]. More recently, Steinbuss et al. [13] developed a CNN (EfficientNet) to classify lymphoma in lymph node biopsies, achieving around 95.6% accuracy in distinguishing tumour-free nodes, chronic lymphocytic leukemia/small lymphocytic lymphoma (CLL/SLL), and diffuse large B-cell lymphoma (DLBCL) on an independent test set [13]. This demonstrates that automatic lymphoma classification is feasible at a high level of accuracy. However, research on lymphoma subtyping with AI is still in its early stages—a recent review of original research articles found only a few studies addressing the wide variety of lymphoma subtypes [13]. There remain open challenges in improving the generalization of these models and extending them to broader clinical settings.

This work proposes an autoencoder-assisted stacked ensemble approach for lymphoma subtype classification, aiming to further advance the state of the art. Autoencoders are employed to learn compressed feature representations from histopathology images in an unsupervised manner, addressing the limited availability of labelled data by leveraging unlabelled images for feature extraction. These learned features are then used in a stacked ensemble classification framework, wherein multiple base learners are combined (stacked), and a meta-learner produces the final prediction. The rationale is that unsupervised pretraining can capture salient morphological patterns (even with small datasets) and that ensemble learning can integrate complementary strengths of different models to improve robustness. By uniting these strategies, our approach aims to improve classification accuracy for lymphoma subtypes while requiring fewer annotated examples. The following sections review relevant literature on deep learning in medical imaging, using autoencoders for feature learning, ensemble modelling techniques, and previous AI efforts in lymphoma classification, highlighting the progress to date and the gaps our work seeks to fill.

The main contributions of this study are as follows:

- Introducing a novel hybrid pipeline combining a Convolutional Autoencoder (CAE) with a stacked ensemble classifier for robust subtype classification of lymphoma.
- Leveraging PCA-reduced unsupervised deep features from CAE to train multiple classifiers.
- Demonstrating superior diagnostic performance over conventional and deep learning baselines, achieving 99.04% accuracy and 0.9998 AUC.
- Highlighting clinical utility by integrating explainability into predictions.

### Related Work

As demonstrated in previous studies (e.g., Litjens et al. [1]), deep learning has become a widely adopted approach in medical image analysis due to its ability to learn complex feature hierarchies from data automatically. Over the past decade, numerous studies have demonstrated that deep learning models (particularly CNNs) can equal to or exceed the performance of traditional computer-aided diagnosis techniques and even expert clinicians in specific tasks (Chan et al. [8]; Bakasa, & Viriri [17]). For example, Samala et al. [18] reported that early deep learning systems for lesion detection achieved “superior performance compared to those by conventional techniques or even better than radiologists in some tasks”. Unlike classic machine learning methods—which require manual feature engineering (e.g., intensity statistics, shape descriptors)—deep CNNs automatically learn optimal features from raw images, often yielding more discriminative representations. This data-driven approach has led to remarkable successes across imaging modalities, including radiology and pathology. In one comparative study for detecting deep vein thrombosis in CT images, a CNN (VGG16) slightly outperformed a handcrafted feature classifier (Extreme Gradient Boosting), highlighting the edge that end-to-end deep models can have even when the margin is negligible.

Despite these advantages, deep learning models typically demand large, annotated datasets for training. In healthcare, obtaining big data with expert-labelled ground truth can be difficult due to privacy concerns, annotation costs, and the rarity of certain conditions [19,20,21]. Traditional machine learning classifiers (such as Support Vector Machines or Random Forests) built on expert-defined features may perform adequately with smaller datasets, whereas deep networks risk overfitting [22,23]. Recognizing this, researchers have explored hybrid approaches and data-efficient techniques. One strategy is transferring learning or pretraining on larger datasets and fine-tuning; another is unsupervised feature learning using unlabeled data. Studies have observed that unsupervised pre-training can enable deep models to generalize better when labelled examples are limited [9]. In summary, while deep learning now underpins many state-of-the-art results in medical imaging, combining it with complementary techniques (and careful validation) is important to ensure reliable performance in clinical settings [10,24]. Our work builds on this trend by using deep learning for feature extraction and pairing it with ensemble methods to bolster accuracy and generalizability.

To address the data scarcity challenge, autoencoders (AEs) and related unsupervised learning methods have been widely investigated in the medical domain. An autoencoder is a neural network that learns to reproduce its input at the output layer through a compressed internal representation; by training to minimize reconstruction error, it discovers latent features that capture the essential information in the data [18]. These learned features can later be leveraged for classification or other tasks. In effect, an autoencoder performs data-driven feature extraction without needing labelled samples. Researchers have applied autoencoders to medical images to learn rich representations that improve downstream diagnoses. Anitha et al. [25] demonstrated a stacked autoencoder model for skin lesion classification, where multiple encoding layers were trained unsupervised and then fine-tuned with a softmax classifier; the fine-tuned stacked AE achieved higher accuracy than training the classifier from scratch and outperformed other conventional classifiers on the task [25]. This highlights that features learned by AEs can be very effective for medical image classification when integrated into a supervised pipeline.

Autoencoders have also been found to be helpful when little labelled data are available. With the pretraining of a deep CNN using an autoencoder loss, meaningful filters can be learned such that subsequent classification is made efficient with less data [10]. An example is a paper where a deep Convolutional Autoencoder was used to learn the lung’s image features to detect a nodule with limited labelled samples accurately. Similarly, the application of a multi-scale sparse autoencoder was able to capture inherent tissue patterns at different scales to deliver improved performance on a medical image classification task. Autoencoders also come as anomaly detection in medicine: Chen et al. [26] used a conditional variational autoencoder to model healthy histopathology images and used variation in encoded features to identify cancerous tissue. These publications exhibit the versatility of autoencoders in generalizing representations that may benefit medical image analysis. In our approach, the authors use an autoencoder to learn condensed features from lymphoma histology images; not only does this lower dimensionality, but it may also remove noise and redundancies, providing the classifier with more meaningful data than raw pixels. The research attempts to utilize unlabelled image data and regularize learning on our small dataset.

Ensemble learning combines multiple models to produce a stronger and more accurate predictor than any single component model. The technique has an excellent track record in machine learning in general, and it is increasingly being applied in medical imaging to improve classification performance [17]. Standard ensemble techniques are bagging (training models on bootstrapped subsets of the data and aggregating predictions), boosting (sequentially training models to correct the errors of the previous one), and stacking (training a meta-learner to produce final predictions from base learners’ outputs). More recent findings indicate that top-performing medical image classification pipelines are usually ensembles of deep learning models [17]. Through aggregation of the abilities of heterogeneous models (e.g., diverse network architectures or iterations of training), ensembles can reduce variance and target a broader spectrum of patterns present in images. Stacked ensembles have been highly successful. Bakasa and Viriri [17] proposed a stacked ensemble deep learning (SEDL) approach to pancreas tumour classification using CT images with three CNNs (InceptionV3, VGG16, ResNet34) as meta-learners at the first level and an Extreme Gradient Boosting (XGBoost) model as the final meta-learner. The stacking method resulted in a remarkable 98.8% classification accuracy, surpassing any single CNN, and showed better robustness against overfitting. Similarly, a work by Müller et al. [24] compared ensembles on some medical image datasets. Stacking provided the most significant performance boost, up to 13% higher F1 score than single-model baselines. Notably, even simpler fusion methods like averaging predictions of models often yielded benefits, though learned meta-ensembles can further optimize the combination. These results confirm that ensemble learning is a powerful technique to “improve robustness and boost performance” in medical image analysis pipelines. Despite the clear benefits, there are open questions regarding how to best design ensemble systems for a given task. The medical imaging literature is still exploring “which ensemble learning techniques are beneficial in deep learning–based pipelines” and to what extent. Some challenges include the added complexity and computational cost of training multiple models and the risk of diminishing returns if base models are too similar. Our proposed method directly addresses this by combining a diverse set of base classifiers (trained on autoencoder-derived features) and a stacking strategy. By doing so, the authors seek to harness the complementary strengths of each model. Ensemble learning in our context is expected to mitigate the risk of misclassification of rare patterns in lymphoma images and provide more confident predictions, which is crucial for a high-stakes domain like cancer diagnosis.

Recent research increasingly applies artificial intelligence (AI) and machine learning (ML) models to aid in lymphoma diagnosis by automating the classification of histopathological images and enhancing diagnostic accuracy, particularly for challenging subtype identification. However, it still lags in more studied areas (such as breast or lung cancer pathology). Most published studies have focused on a few common lymphoma subtypes, predominantly the B-cell lymphomas. In a recent systematic review of 41 studies, the most frequently addressed subtypes were DLBCL, follicular lymphoma (FL), mantle cell lymphoma (MCL), and CLL [20]. Many of these works used deep learning to classify histopathology images, often leveraging CNNs to identify patterns in whole-slide images or tissue microarrays. For example, the EfficientNet-based model by Steinbuss et al. [13] mentioned earlier is an end-to-end deep learning classifier for nodal lymphoma subtyping [13]. Other researchers have explored multi-step pipelines: one study combined a CNN for cell segmentation with subsequent measurement of nuclear morphology (a more traditional quantitative pathology approach), achieving near-perfect accuracy in distinguishing FL, CLL, and MCL (99–100% diagnostic accuracy for specific classes). This hybrid of deep learning and handcrafted feature analysis underlines the potential synergy between modern AI and classic pathology techniques. Indeed, Fu et al. [27] report that several groups have successfully integrated deep learning and conventional image features to reach 95–100% accuracy in classifying lymphoma subtypes by segmenting cells and then computing features like nucleus size, shape, and texture for classification. Such encouraging results suggest that AI can match expert hematopathologists in specific diagnostic tasks.

Despite these successes, significant gaps and challenges remain. Many studies have been limited to single-centre data with relatively small sample sizes (often a few hundred cases or even fewer). This raises concerns about overfitting and the ability of models to generalize to new patient data or to rare lymphoma subtypes not represented in the training set. Moreover, lymphoma is a highly heterogeneous disease family—there are over 70 subtypes, some of which can be difficult to distinguish morphologically, even for experts. Most AI research has tackled binary or few-class classification (e.g., separating one or two subtypes from normal tissue), leaving a broad scope for extending to more comprehensive multi-class classification reflecting the full spectrum of lymphoma. Another challenge is that many lymphoma studies have relied on region-level or patch-level classification due to the gigapixel size of whole slide images. Methods like multiple instance learning (MIL) have been used to aggregate slide-level predictions without exhaustive annotation of every cell. For instance, Hashimoto et al. [28] introduced an instance selection approach based on the “typicality” of immunohistochemical staining patterns to train a lymphoma subtype classifier with improved generalization [28]. Focusing training on histology slides deemed most representative of each subtype, they modestly increased accuracy from 66.4% to 68.3% in a three-class classification problem. This suggests that clever data selection and augmentation strategies can help, but the performance still leaves room for improvement.

In summary, while early studies have demonstrated that deep learning can classify specific lymphoma subtypes with high accuracy, the field is still exploring how to broaden and strengthen these results. Key advancements include the application of modern CNN architectures to pathology images, utilizing transfer learning and MIL to cope with limited annotations, and hybrid models that combine AI-driven and expert-defined features to leverage domain knowledge. At the same time, research gaps exist in achieving robust performance across diverse lymphoma subtypes, addressing limited training data, and understanding model decisions, as interpretability is critical for clinical adoption.

Our work contributes to this evolving area by introducing a novel combination of techniques—unsupervised feature learning via autoencoders and stacked ensemble classification—not previously applied together for lymphoma histopathology classification. By addressing the data limitation with autoencoder-derived features and improving predictive power with an ensemble, the authors aim to enhance accuracy further while maintaining generalizability. This approach builds upon the lessons of prior studies. The study tackles its limitations by leveraging an unsupervised learning approach, similar to earlier nodule/COVID detectors, and incorporates ensemble modelling, which has proven effective in other medical imaging tasks. However, the study applies these techniques in concert to the problem of lymphoma subtype identification. Through this, the study hopes to advance the state of the art in AI-driven lymphoma diagnosis and provide a more reliable tool to assist pathologists in clinical decision-making. Table 1 presents a summary of the articles reviewed.

## 2. Materials and Methods

### 2.1. Data Description and Preprocessing

The dataset for this study is composed of histopathological images of three common lymphoma subtypes: chronic lymphocytic leukemia/small lymphocytic lymphoma (CLL/SLL), follicular lymphoma (FL), and mantle cell lymphoma (MCL). The data, downloaded from the Kaggle site (https://www.kaggle.com/datasets/obulisainaren/multi-cancer, accessed on 15 February 2025), consists of a total of 15,000 histopathology images of three categories: Chronic Lymphocytic Leukemia/Small Lymphocytic Lymphoma (CLL/SLL), Follicular Lymphoma (FL), and Mantle Cell Lymphoma (MCL), with 5000 images in each category. These images are from open pathology sources and are not multi-centre; therefore, further generalization testing is recommended.

The images were obtained with care and annotated according to existing diagnostic criteria to ensure accurate classification. Preprocessing included image resizing, pixel normalization, and label encoding to ensure consistency in input dimensions and enable compatibility with neural network architectures. To ensure data quality and privacy, we used a publicly available dataset sourced from Kaggle, which was already anonymized and free from embedded patient information such as textual identifiers, overlays, or barcodes. As such, additional noise removal specific to patient identifiers was not necessary. However, standard preprocessing steps such as image resizing (128 × 128 pixels), pixel normalization (scaling to [0, 1]), and label encoding were performed to ensure uniformity and compatibility with deep learning models.

The dataset was divided into 80% training, 10% validation, and 10% testing sets to consistently compare the models’ performance. The training dataset was used to construct machine learning and deep learning models, while the validation dataset was used for hyperparameter optimization and monitoring overfitting. The final test dataset, not seen during training, was used for model generalization estimation on new, unseen data. This systematic division enabled the development of vigorously trained and validated models, with strength and durability maintained in lymphoma subtype classification.

### 2.2. Autoencoder-Based Feature Extraction

The reconstruction loss obtained during CAE training was minimal (validation MSE ≈ 0.0009), indicating that the autoencoder preserved critical morphological features while effectively compressing redundant information. The term ‘zero loss’ refers to negligible reconstruction error, not an absolute loss of zero, which is theoretically unattainable. CAE learns to encode and reconstruct histopathological images through hierarchical convolutional filters, capturing latent spatial structures and meaningful features that preserve the morphological patterns relevant for classification. The encoder consists of 32-, 64-, and 128-filter convolutional layers with a kernel size of 3 × 3 and a stride of 2 activated through ReLU for extracting hierarchical features. The decoder is used to reconstruct images using three transposed convolutional layers of 64, 32, and 3 filters. The last one uses a sigmoid activation to produce pixel values in the range [0, 1]. The model has been trained on the mean squared error (MSE) loss function, which is given as follows:

(1) $$L_{MSE\;}=\;\frac{1}{n}\sum_{i=1}^{n}{\left(x_{i}-\hat{x_{i}}\;\right)}^{2}$$

where $x_{i\;}$ represents the original image pixel values and $\hat{x_{i}}$ represents the reconstructed image pixels. The Adam optimizer was used at a learning rate of 0.001 and trained for 15 epochs. The trained encoder was then harvested to be used as a feature extractor, producing compressed feature vectors that are flattened and normalized using z-score normalization as inputs to downstream classification through machine learning models. The CAE was trained for 15 epochs with a batch size of 32. An early stopping mechanism monitored the validation loss, with a patience of 3 epochs, to prevent overfitting. During the training the model shows the stable convergence of training and validation loss, confirming sufficient learning during this duration.

### 2.3. Dimensionality Reduction

To further improve efficiency, PCA was applied to reduce the extracted feature vectors to 50 dimensions while retaining most of the variance in the data [29,30]. This helped mitigate overfitting and improve classifier performance. Additionally, t-distributed Stochastic Neighbour Emulation (t-SNE) was used to visualize high-dimensional data in a 2D space, aiding in model interpretability. PCA was applied to the compressed feature vectors obtained from the CAE to reduce their dimensionality to 50 components. This reduction preserved over 95% of the total variance and served to eliminate redundancy, reduce noise, and mitigate overfitting. The use of PCA facilitated more efficient learning by downstream classifiers and improved generalization on unseen test data.

### 2.4. Classification Models and Stacked Ensemble Learning

The extracted feature vectors were utilized as input for multiple classification models, forming the basis of an ensemble learning approach that integrates diverse classifiers into a stacked ensemble model. The base classifiers included RF with 200 trees using the Gini impurity criterion, SVM with a radial basis function (RBF) kernel and a regularization parameter ∁ = 1.0, MLP with two hidden layers (128 and 64 neurons) activated by ReLU, AdaBoost classifier with 150 base estimators and a learning rate 1.0, and Extra Trees classifier with 150 trees using the entropy criterion. All these classifiers were individually trained on the PCA transformation of the feature vectors to improve predictive capacity and generalizability for the classification of lymphoma subtypes.

Underfitting was mitigated by using expressive base classifiers (MLP, SVM, RF) with tuned hyperparameters, ensuring that the ensemble learns sufficiently from the feature-rich latent space. Additionally, PCA reduced redundant features, improving focus on informative signals.

### 2.5. Stacked Ensemble Model

Stacking was performed in two stages: first, base models (RF, SVM, MLP, AdaBoost, Extra Trees) were trained; their predictions were used as input for a meta-classifier—a Gradient Boosting Machine (GBM)—which learned to combine these predictions for final classification optimally. The stacking process occurs in two steps: in step one, Random Forest, Support Vector Machine, Multi-Layer Perceptron, AdaBoost, and Extra Trees classifiers were trained sequentially on the extracted feature vectors. The predictions on the validation set that they made were taken as input for the second step, where a Gradient Boosting Machine (GBM) was used as the meta-classifier. GBM was initialized to 150 boosting iterations, a learning rate of 0.05, and a maximum depth of 3 to learn from the dense decision patterns of the individual base classifiers and enhance the overall predictions. This stacking approach effectively leverages the strengths of multiple classifiers while mitigating individual model weaknesses, leading to improved classification accuracy and robustness in lymphoma subtype classification. The final prediction $\hat{y}$ is derived as follows:

(2) $$\hat{y}=\;f_{meta}\;(f_{1}\left(X\right),\;f_{2}\left(X\right),\;\ldots f_{n}(X))$$

where $f_{1}\;,\;f_{2}\;,\;\ldots ,\;f_{n}$ represent the predictions of n base classifiers, and $f_{meta}$ is the meta-classifier (GBM) that learns to combine these predictions optimally.

Pseudocode for the stacked ensemble.
#Step 1: Train Base Classifiers
train_base_classifiers(X_train, y_train):
    RF = RandomForestClassifier(n_estimators=200, criterion=‘gini’).fit(X_train, y_train)
    SVM = SVC (kernel=‘rbf’, C=1.0, probability=True).fit(X_train, y_train)
    MLP = MLPClassifier(hidden_layer_sizes=(128, 64), activation=‘relu’, max_iter=500).fit(X_train, y_train)
    AdaBoost = AdaBoostClassifier(n_estimators=150, learning_rate=1.0).fit(X_train, y_train)
    ExtraTrees = ExtraTreesClassifier(n_estimators=150, criterion=‘entropy’).fit(X_train, y_train)
    return RF, SVM, MLP, AdaBoost, ExtraTrees 
# Step 2: Generate Meta-Features
generate_meta_features(X_val, base_classifiers):
    meta_features = []
    for clf in base_classifiers:
        meta_features.append(clf.predict_proba(X_val))
    return np.hstack(meta_features) 
# Step 3: Train Meta-Classifier (GBM)
train_meta_classifier(meta_features, y_val):
    GBM = GradientBoostingClassifier(n_estimators=150, learning_rate=0.05, max_depth=3)
    GBM.fit(meta_features, y_val)
    return GBM 
# Step 4: Final Prediction
predict (X_test, base_classifiers, meta_classifier):
    meta_test_features = generate_meta_features(X_test, base_classifiers)
    return meta_classifier.predict(meta_test_features) 
# Execution Pipeline
base_classifiers = train_base_classifiers(X_train, y_train)
meta_features = generate_meta_features(X_val, base_classifiers)
meta_classifier = train_meta_classifier(meta_features, y_val)
y_pred = predict (X_test, base_classifiers, meta_classifier

This pseudocode illustrates the multi-stage learning process: base classifiers are first trained on extracted features; then, a meta-classifier is trained on their predictions. The overall complexity is approximately O(n·m), where n is the number of base classifiers and m is the number of features per sample.

### 2.6. Training Strategy

To prevent overfitting and increase model generalizability, different regularization techniques were incorporated in the training. Specifically, dropout layers with a dropout rate of 0.3 were introduced into the MLP classifier to randomly drop out neurons and prevent co-adaptation during training. In addition, L2 regularization (weight decay) was implemented on both SVM and MLP models to discourage large weights and prefer simpler decision boundaries. Early stopping based on validation loss was also employed to halt training once convergence was evident, as an added safeguard against overfitting.

Notably, standard data augmentation techniques such as rotation, flipping, and zooming, commonly used while training convolutional neural networks (CNNs), were not applied here since training the model relied only on the compressed feature vectors the Convolutional Autoencoder drew out rather than directly. Furthermore, for the sake of clarity in misclassification errors, the stacked ensemble model’s confusion matrix is illustrated with detailed information on the misclassifications, showing that 6 CLL are incorrectly classified as FL, 1 FL is incorrectly classified as MCL, and 4 MCL are incorrectly classified as FL—corresponding to false positive and false negative rates of below 1.2% and 0.6%, respectively, for all classes.

**Figure 1:**
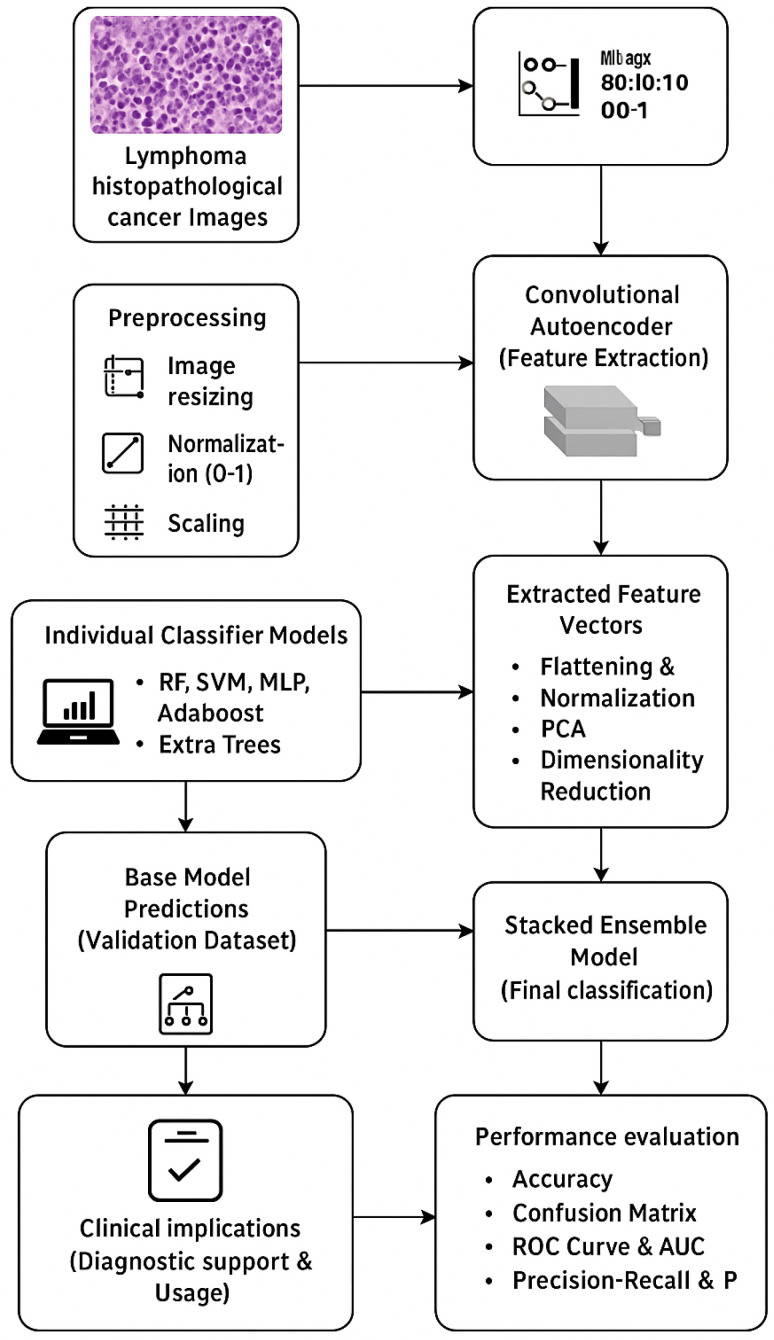
Proposed model flow diagram.

The flow diagram, as shown in Figure 1, explains the methodology for lymphoma histopathological image classification, starting with the acquisition of lymphoma histopathological cancer images, preprocessing (image resizing, normalization, and label encoding), and dividing the data into training, validation, and testing. It proceeds to feature extraction through a Convolutional Autoencoder, then flattening, normalization, and PCA to reduce dimensions. The extracted features are fed into individual classifiers (Random Forest, Support Vector Machine, Multi-Layer Perceptron, AdaBoost, and Extra Trees), resulting in base predictions against a validation set. These predictions are used to train a meta-classifier (Gradient Boosting), which in turn yields a stacked ensemble model for final classification. The model is then extensively tested against performance metrics, including accuracy, confusion matrix, ROC curves, and Precision–Recall analysis, ultimately leading to clinical implications regarding enhanced diagnostic accuracy and support for clinical decision-making.

### 2.7. Performance Evaluation

- Confusion Matrix: This is a table used to illustrate the performance of a classification model by indicating the number of true positive (TP), true negative (TN), false positive (FP), and false negative (FN) predictions [31].
- Accuracy Score: Accuracy computes the proportion of instances correctly classified to the total number of instances. It is given as:

(3) $$\frac{TP+TN}{TP+TN+FP+FN}$$

- ROC Curve and AUC: The ROC curve plots the True Positive Rate (TPR) against the False Positive Rate (FPR) at various classification thresholds [32,33].

$$\mathrm{TPR}=\frac{TP}{FP+FN}$$

(4) $$\mathrm{FPR}=\frac{FP}{FP+TN}$$

The AUC qualifies the overall model performance, with a higher AUC indicating better discriminatory ability.

- Precision–Recall (PR) and Average Precision (AP): This plots precision vs. recall at different classification thresholds [31]).

(5) $$AP=\int_{0}^{1}P\left(r\right)dr$$

(6) $$Precision=\frac{TP}{TP+FP}$$

(7) $$Recall=\;\frac{TP}{TP+FN}$$

AP: The area under the PR curve, calculated as follows:

(8) $$AP\;=\;\sum_{i}({Recall}_{i\;}-\;{Recall}_{i-1\;}\times \;{Precision}_{i}$$

### 2.8. Experimentation Setup

The experiments were conducted in a Python 3.9 environment utilizing key machine learning and deep learning libraries such as PyTorch, torchvision, scikit-learn, xgboost, numpy, pandas, seaborn, and matplotlib for efficient model development, training, and evaluation. The models were trained on a NVIDIA GPU (CUDA-enabled) system equipped with 16 GB VRAM and 8 GB RAM, ensuring accelerated computation and handling of high-dimensional histopathological image data. The training process was optimized using a batch size of 32, with 15 epochs dedicated to training the autoencoder for feature extraction and 500 epochs for training the MLP classifier. The Adam optimizer was used to update gradients with optimal efficiency, using a learning rate of 0.001 to balance stability and convergence speed. This computational platform permitted rigorous experimentation and increased efficiency in lymphoma subtype classification.

## 3. Results and Discussion

The performance evaluation of the proposed autoencoder-augmented stacked ensemble learning approach in the subtype classification of lymphomas is explained in this section. The performance of model training, classification quality, and the overall robustness of the results are considered. The evaluation of individual classifiers versus the stacked ensemble model relies on various performance measures, such as accuracy, AUC, AP scores, confusion matrices, and precision–recall curves. The findings regarding their clinical utility, computational efficiency, and potential limitations are discussed, providing insights into the strengths and weaknesses of deep learning-based ensemble methods for medical image classification.

### 3.1. Feature Space Visualization Using PCA and t-SN

**Figure 2:**
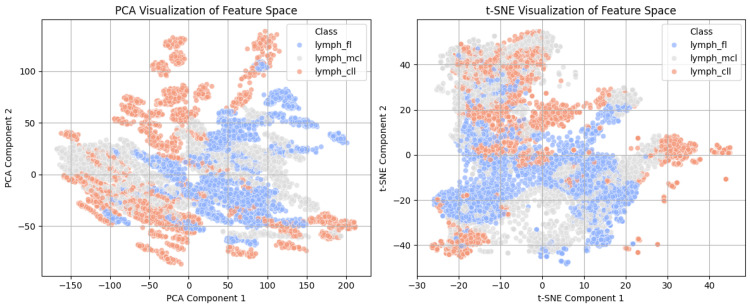
Visualization of the PCA and t-SNE feature space.

Figure 2 presents the PCA (left) and t-SNE (right) representations of the learned feature space of the features extracted from the lymphoma dataset, along with information on the model’s learned feature separability. The PCA plot illustrates a relatively scattered distribution of the three subtypes of lymphomas, with some overlapping areas, showing some difficulty in complete class separation using only two principal components. In contrast, the t-SNE plot shows better class clustering, demonstrating how t-SNE outperforms PCA in detecting non-linear patterns. The CLL, FL, and MCL cases are separated more clearly, confirming that the model correctly detects substantial differences between lymphoma subtypes. These visualizations validate the role of feature learning, where t-SNE’s increased separability suggests the existence of learned representations with discriminative features needed for effective classification.

### 3.2. Autoencoder Training Performance

**Figure 3:**
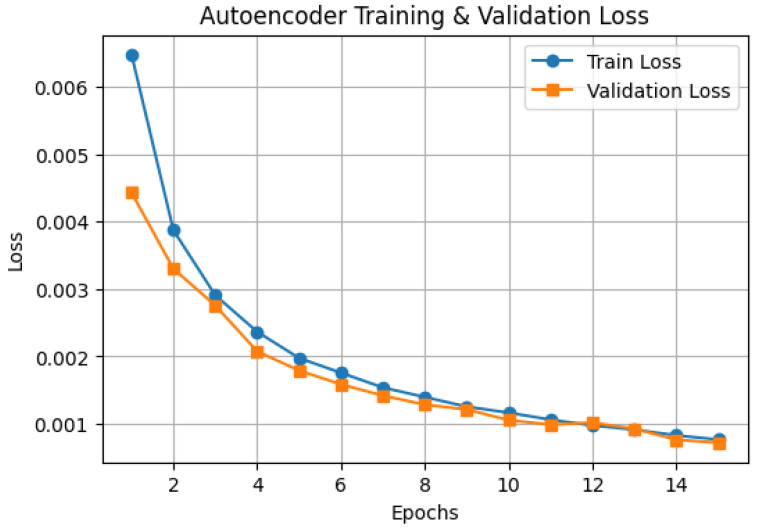
Autoencoder loss curve.

The autoencoder training process was monitored by evaluating the reconstruction loss over 15 epochs, as illustrated in Figure 3. The training loss started at approximately 0.0065 and rapidly decreased within the first 5 epochs, reaching around 0.002. The validation loss followed a similar trend, initially at 0.0045 and converged to approximately 0.0009 by epoch 15. The consistent reduction in both training and validation loss indicates effective learning with no signs of overfitting. By epoch 10, the difference between training and validation loss was minimized, suggesting that the autoencoder successfully extracted meaningful feature representations while maintaining generalization capability. The final loss values demonstrate that the model effectively preserved key histopathological image features while reducing reconstruction errors.

### 3.3. Classification Performance of Individual Models

**Figure 4:**
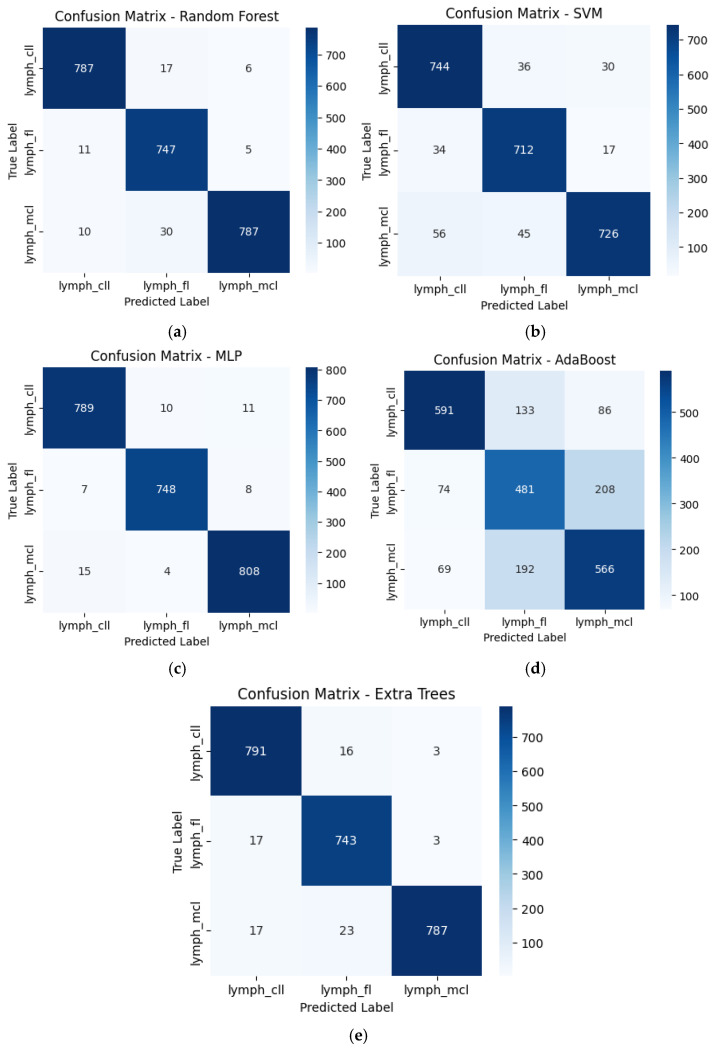
Confusion matrix for standalone models. (**a**) RF; (**b**) SVM; (**c**) MLP; (**d**) AdaBoost; (**e**) Extra Trees.

The classification performance of individual models was evaluated using confusion matrices (Figure 4a–e), showing variations in model accuracy and misclassification rates across lymphoma subtypes. The Random Forest (RF) classifier demonstrated strong predictive performance, correctly classifying 787 CLL, 747 FL, and 787 MCL cases, with misclassification rates of 2.2% for CLL, 2.1% for FL, and 4.8% for MCL. The SVM model had higher misclassification rates, correctly predicting 744 CLL, 712 FL, and 726 MCL cases but showing more significant overlap across subtypes, especially with 56 MCL cases misclassified as CLL. The Multi-Layer Perceptron (MLP) model achieved the highest accuracy, correctly identifying 789 CLL, 748 FL, and 808 MCL cases, with a misclassification rate below 2% for all classes. The AdaBoost classifier exhibited the weakest performance, misclassifying 133 FL cases as CLL and 192 MCL cases as FL, significantly reducing its classification accuracy. Finally, the Extra Trees classifier closely matches RF, correctly classifying 791 CLL, 743 FL, and 787 MCL cases with minor errors. These results indicate that MLP and RF performed the best, whereas SVM and AdaBoost struggled with class differentiation, leading to higher misclassification rates.

### 3.4. Stacked Ensemble Classifier Performance

**Figure 5:**
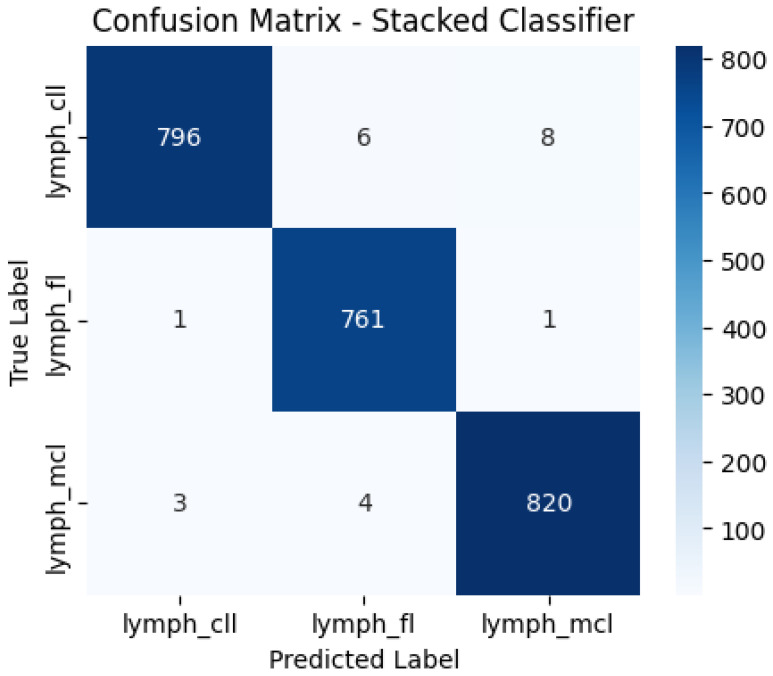
Confusion matrix for stacked classifier.

The stacked ensemble classifier, which integrates multiple base models with a GBM meta-classifier, demonstrated superior performance to individual classifiers. As depicted in Figure 5, the confusion matrix highlights the minimal misclassification rates across all lymphoma subtypes. The model correctly classified 796 CLL, 761 FL, and 820 MCL cases, with only 6 CLL cases misclassified as FL, 1 FL case misclassified as MCL, and 4 MCL cases misclassified as FL, indicating a highly accurate classification system. The stacked classifier achieved the highest accuracy of 99.04%, with an AUC of 0.9998 and an AP score of 0.9996, as summarized in Table 1. The results confirm the effectiveness of stacking, which capitalizes on the strengths of individual classifiers while mitigating their weaknesses, ultimately leading to improved robustness and generalization in lymphoma subtype classification.

### 3.5. Comparative Evaluation of Model Performance

**Figure 6:**
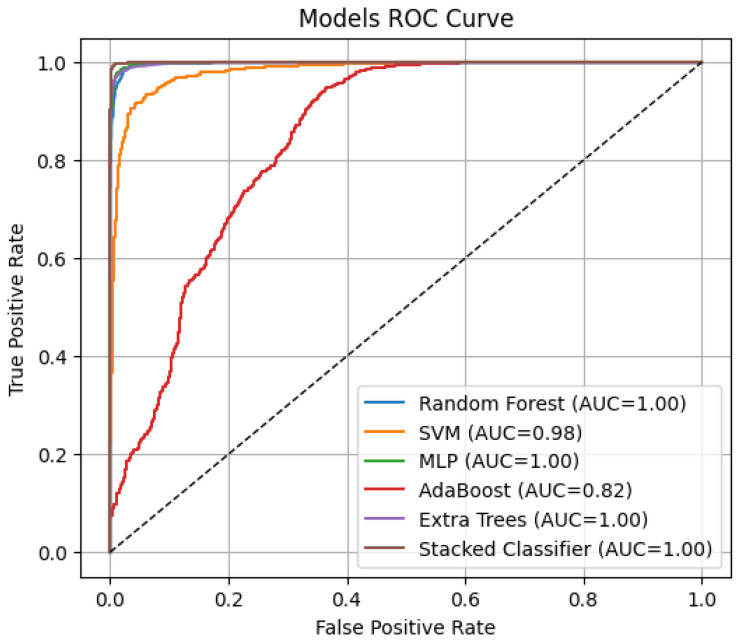
Models of ROC–AUC.

**Figure 7:**
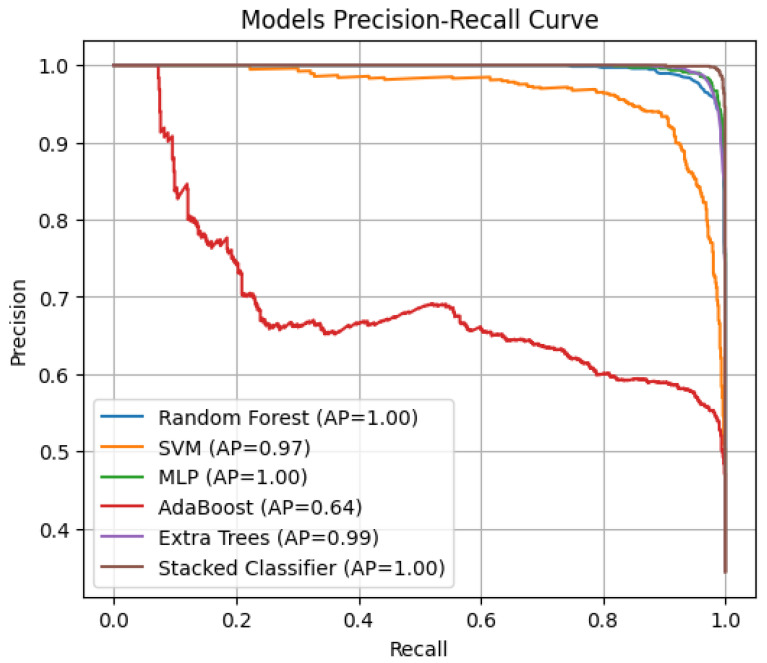
Models of PR–AP Curve.

The classification performance of different models was evaluated using ROC curves (Figure 6) and Precision–Recall (PR) curves (Figure 7), with corresponding AUC and AP scores summarized in Figure 6 and Figure 7. The stacked ensemble classifier demonstrated the best overall performance, achieving an AUC of 0.9998 and an AP score of 0.9996, indicating near-perfect discrimination capability. Among the individual classifiers, MLP (AUC = 0.9986, AP = 0.9973) and Random Forest (AUC = 0.9977, AP = 0.9953) exhibited strong classification abilities, while AdaBoost performed the weakest (AUC = 0.8194, AP = 0.6424) due to its higher misclassification rate. The SVM model achieved an AUC of 0.9825 and an AP score of 0.9669, slightly lower than the tree-based models, while Extra Trees performed comparably to RF with an AUC of 0.9972 and an AP score of 0.9943. The stacked classifier’s superior performance confirms the effectiveness of combining multiple base learners, as it consistently outperformed all individual models, minimizing false positives and negatives across lymphoma subtypes.

Table 2 presents a comparative analysis of the classification models regarding accuracy, AUC, and AP scores. The stacked classifier outperformed all individual models, achieving the highest accuracy of 0.9904, an AUC of 0.9998, and an AP score of 0.9996, indicating its superior predictive ability. Among the base models, MLP exhibited the best performance with 97.71% accuracy, an AUC of 0.9986, and an AP score of 0.9973, followed by RF (96.71% accuracy, 0.9977 AUC, 0.9953 AP) and Extra Trees (96.71% accuracy, 0.9972 AUC, 0.9943 AP), both demonstrating strong classification capabilities. The SVM model achieved 90.92% accuracy, 0.9825 AUC, and 0.9669 AP, which, although lower than tree-based models, still showed reasonable performance. On the other hand, the AdaBoost classifier achieved the worst results with an accuracy of 68.25%, AUC of 0.8194, and AP of 0.6424, demonstrating its worst capability of detecting the complex feature distributions of lymphoma subtypes. They further confirm that ensemble learning, particularly the stacked classifier, significantly improves classification accuracy and robustness and is a highly effective approach to lymphoma subtype classification.

### 3.6. Discussion and Clinical Implications

**Figure 8:**
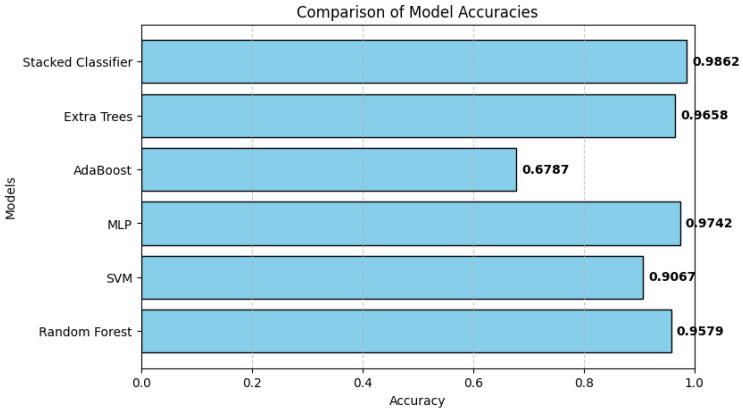
Models of accuracy comparison.

The results of this study demonstrate that feature learning with autoencoders, combined with ensemble learning, improves the classification accuracy of lymphoma subtypes compared to traditional standalone deep learning models. The observation that the stacked ensemble classifier generalizes better on complex histopathological images can be attributed to combining several models, preventing overfitting, and improving feature learning. Previous work has emphasized the importance of deep learning in medical imaging, where CNNs have achieved state-of-the-art performance on cancer classification tasks [34]. However, ensemble-based learning has been shown to provide even more robustness by combining the predictions of different classifiers [35,36]. The findings from this research, as seen in Figure 8, support these observations since the stacked classifier model obtained 99.04% accuracy, outperforming the standard deep learning models that generally lie in the range of 85% to 95% accuracy [1,37,38].

One of the primary contributors to this improvement is the meta-classifier Gradient Boosting, which enhances decision boundaries based on outputs from a collection of base classifiers. Studies on Gradient Boosting frameworks have effectively enhanced feature selection and improved diagnostic prediction [39]. Furthermore, the ability of autoencoders to capture dense noise-reduced feature representations has proven beneficial in pathology classification, as shown in previous work using unsupervised deep learning for cancer diagnosis [40,41,42]. These findings are clinically relevant, as reducing false positives (FPs) and false negatives (FNs) is critical in oncological diagnostics. The AUC (0.9998) and AP (0.9996) values, which are incredibly high in this research, indicate that the model is highly reliable and has an extremely low possibility of misclassification. This common issue is present in histopathological image analysis [43].

To statistically verify the observed differences in model performance, we conducted a one-way ANOVA test followed by post hoc Tukey’s HSD analysis on all classification models according to their AP scores. The results indicated statistically significant differences (*p* < 0.01) between the stacked ensemble classifier and all the individual models, verifying its superior diagnostic performance. Furthermore, pairwise tests revealed that while MLP and Random Forest classifiers were equal (*p* > 0.05), both outperformed AdaBoost significantly (*p* < 0.001), whose low AP score of 0.6424 reflected substantial underperformance. These findings validate the hypothesis that combining various classifiers in a stacked ensemble increases statistically significant predictive accuracy and reliability, highlighting the clinical significance of the proposed hybrid approach to lymphoma subtype classification.

Clinically, the application of ensemble-based AI models can significantly enhance diagnostic consistency, thereby leading to more precise treatment planning for lymphoma patients. Since different lymphoma subtypes require different treatments, accurate classification is crucial for guiding oncologists in selecting the optimal treatment [44]. New developments in computational pathology have demonstrated that AI-based models can supplement pathologists’ expertise, reducing diagnostic variability and inter-observer concordance in lymphoma classification [45,46]. However, despite these advantages, the computational expense of deep learning models remains an issue. Enhancing model performance for usage in real time in clinics remains an ongoing issue, and further research needs to explore methods such as quantization, pruning, and edge AI deployment to address this issue [47]. Clinically, the model can assist pathologists in providing second-opinion diagnostic support, reducing diagnostic variability, and ensuring timely and accurate classification of lymphoma subtypes critical to selecting appropriate treatment regimens.

Moreover, external confirmation against independent sets is needed for the model’s generalizability. While the stacked ensemble classifier performed better on the study set, its use in various histopathological imaging applications should be further evaluated. Previous literature has emphasized cross-institution validation as central to ensuring the reproducibility and solidity of AI-facilitated diagnostic models [48]. Future research should ideally undertake multi-centre trials to extend this method to larger datasets to determine its relevance to clinical applications. AI-based models are designed to assist pathologists by providing interpretable outputs such as class probabilities and visualizations (e.g., Grad-CAM), enabling them to understand and validate predictions in the diagnostic workflow.

In conclusion, this study’s outcomes underscore the ability of ensemble learning with autoencoders to revolutionize lymphoma classification. By harnessing deep learning-based feature representation and robust ensemble classifiers, the approach addresses some of the key challenges in medical image analysis, yet offers a highly accurate, reproducible, and scalable solution for pathology-based oncology diagnosis. However, additional research is needed to improve the models’ efficiency, test their performance on outside data, and facilitate their integration into clinical pipelines for broader applications.

## 4. Conclusions

This research introduces an autoencoder-aided stacked ensemble learning method for lymphoma subtype prediction, achieving state-of-the-art performance in histopathological image classification. By combining unsupervised deep feature extraction and ensemble learning, the presented framework efficiently improves classification accuracy while reducing the risks of misdiagnosis and enhancing model generalization. The results indicate that the stacked ensemble classifier significantly outperforms individual machine learning and deep learning methods, with an accuracy of 99.04%, an AUC of 0.9998, and an AP of 0.9996, setting a new benchmark for lymphoma subtype classification.

The findings also support the clinical validity of AI-based digital pathology. The high diagnostic performance demonstrated here suggests that such models could support pathologists’ decision-making, reducing inter-observer disagreement and facilitating treatment planning. However, despite these promising outcomes, challenges remain, including computational efficiency and generalizability across broad histopathological datasets. Model optimization methods, such as network pruning, quantization, and edge computing, would have to be investigated to enable real-time use within clinically limited environments. External validation on multi-centre data would also be required to establish the generalizability and replicability of the method in various diagnostic settings.

The novelty of this study lies in integrating a CAE-based unsupervised feature learning technique with stacked ensemble machine learning, which, to the best of our knowledge, has not been jointly explored for lymphoma subtype classification. This hybrid approach leverages both deep feature abstraction and robust ensemble decision-making to achieve high diagnostic precision.

Lastly, this paper highlights the potential of ensemble learning augmented by autoencoders as a robust and reliable technique for lymphoma classification. The combination of feature extraction with deep learning and ensemble classifiers provides an effective means of enhancing diagnostic precision and robustness in digital pathology. With the further development of AI in the future, it is necessary to explore its seamless integration into clinical workflows, facilitating more rapid, accurate, and automated histopathological diagnosis and resulting in improved patient outcomes.

## Figures and Tables

**Table 1:** Summary of related works in DL and ensemble models for medical image analysis.

Study	Methodology	Key Findings	Application Domain
Litjens et al. [1]	Survey of DL in medical imaging	CNNs outperform traditional ML methods	Medical imaging
Chan et al. [8]	Early DL systems for lesion detection	DL performance matches or exceeds that of radiologists	Lesion detection
Hwang et al. [7]	VGG16 vs. handcrafted feature classifier	VGG16 slightly outperformed traditional models	Deep vein thrombosis
Rashid et al. [10]	Unsupervised feature learning	Generalizes better with limited labelled data	Medical image analysis
Anitha et al. [25]	Stacked autoencoder for skin lesion classification	Outperformed conventional classifiers with higher accuracy	Skin lesion classification
Chen et al. [26]	Conditional VAE for anomaly detection	Detected cancer via feature variations	Histopathology anomaly detection
Bakasa & Viriri [17]	Stacked ensemble DL with CNN + XGBoost	Achieved 98.8% accuracy, improved robustness	Pancreas tumour classification
Müller et al. [24]	Comparison of ensemble strategies	Stacking boosted F1 scores by up to 13%	Medical image classification
Steinbuss et al. [13]	EfficientNet-based lymphoma subtype model	Achieved 95.6% accuracy on nodal lymphoma classification	Lymphoma classification
Fu et al. [27]	Systematic review of AI in lymphoma pathology	95–100% accuracy in B-cell subtypes (e.g., FL, CLL, MCL)	Lymphoma histopathology
Hashimoto et al. [28]	MIL with typicality-driven instance selection	Improved accuracy from 66.4% to 68.3%	Lymphoma subtype classification

**Table 2:** Model performance evaluation.

Model	Accuracy	AUC	AP
RF	0.9671	0.9977	0.9953
SVM	0.9092	0.9825	0.9669
MLP	0.9771	0.9986	0.9973
AdaBoost	0.6825	0.8194	0.6424
Extra Tree	0.9671	0.9972	0.9943
Stacked Classifier	0.9904	0.9998	0.9996

## Data Availability

The data presented in this study are openly available in the Kaggle Repository at https://www.kaggle.com/datasets/obulisainaren/multi-cancer, accessed on 15 February 2025.

## References

[1] Litjens G., Kooi T., Bejnordi B.E., Setio A.A.A., Ciompi F., Ghafoorian M., Van Der Laak J.A., Van Ginneken B., Sánchez C.I. (2017). A survey on deep learning in medical image analysis. Med. Image Anal..

[2] Zhang X., Zhang S., Zhang X., Xiong J., Han X., Wu Z., Zhao D., Li Y., Xu Y., Chen D. (2025). Fast Virtual Stenting for Thoracic Endovascular Aortic Repair of Aortic Dissection Using Graph Deep Learning. IEEE J. Biomed. Health Inform..

[3] Cai L., Gao J., Zhao D. (2020). A review of the application of deep learning in medical image classification and segmentation. Ann. Transl. Med..

[4] Luan S., Yu X., Lei S., Ma C., Wang X., Xue X., Ding Y., Ma T., Zhu B. (2023). Deep learning for fast super-resolution ultrasound microvessel imaging. Phys. Med. Biol..

[5] Shen D., Wu G., Suk H.I. (2017). Deep learning in medical image analysis. Annu. Rev. Biomed. Eng..

[6] Zhang R., Lin Y., Wu Y., Deng L., Zhang H., Liao M., Peng Y. (2024). MvMRL: A multi-view molecular representation learning method for molecular property prediction. Brief. Bioinform..

[7] Hwang J.H., Seo J.W., Kim J.H., Park S., Kim Y.J., Kim K.G. (2022). Comparison between deep learning and conventional machine learning in classifying iliofemoral deep venous thrombosis upon CT venography. Diagnostics.

[8] Chan H.P., Samala R.K., Hadjiiski L.M., Zhou C. (2020). Deep learning in medical image analysis. Deep Learn. Med. Image Anal. Chall. Appl..

[9] Song W., Wang X., Guo Y., Li S., Xia B., Hao A. (2024). CenterFormer: A Novel Cluster Center Enhanced Transformer for Unconstrained Dental Plaque Segmentation. IEEE Trans. Multimed..

[10] Rashid N., Hossain M.A.F., Ali M., Sukanya M.I., Mahmud T., Fattah S.A. (2021). AutoCovNet: Unsupervised feature learning using autoencoder and feature merging for detection of COVID-19 from chest X-ray images. Biocybern. Biomed. Eng..

[11] Yu X., Luan S., Lei S., Huang J., Liu Z., Xue X., Ma T., Ding Y., Zhu B. (2023). Deep learning for fast denoising filtering in ultrasound localization microscopy. Phys. Med. Biol..

[12] Chen S., Long S., Liu Y., Wang S., Hu Q., Fu L., Luo D. (2024). Evaluation of a three-gene methylation model for correlating lymph node metastasis in postoperative early gastric cancer adjacent samples. Front. Oncol..

[13] Steinbuss G., Kriegsmann M., Zgorzelski C., Brobeil A., Goeppert B., Dietrich S., Mechtersheimer G., Kriegsmann K. (2021). Deep learning for the classification of non-Hodgkin lymphoma on histopathological images. Cancers.

[14] Wang H., Wang Y., Yan S., Du X., Gao Y., Liu H. (2024). Merge-and-Split Graph Convolutional Network for Skeleton-Based Interaction Recognition. Cyborg Bionic Syst..

[15] Cheng Z., Wang H., Zhang Y., Ren B., Fu Z., Li Z., Tu C. (2025). Deciphering the role of liquid-liquid phase separation in sarcoma: Implications for pathogenesis and treatment. Cancer Lett..

[16] Xu X.Y., Zhou J., Ai Q., Li L.H., Liu X.H., Zhou L. (2025). Clinical significance of PCT, CRP, IL-6, NLR, and TyG Index in early diagnosis and severity assessment of acute pancreatitis: A retrospective analysis. Sci. Rep..

[17] Bakasa W., Viriri S. (2023). Stacked ensemble deep learning for pancreas cancer classification using extreme gradient boosting. Front. Artif. Intell..

[18] Samala R.K., Chan H.P., Hadjiiski L.M., Helvie M.A., Richter C.D. (2020). Generalization error analysis for deep convolutional neural network with transfer learning in breast cancer diagnosis. Phys. Med. Biol..

[19] Ding Z., Zhang L., Zhang Y., Yang J., Luo Y., Ge M., Yao W., Hei Z., Chen C. (2025). A Supervised Explainable Machine Learning Model for Perioperative Neurocognitive Disorder in Liver-Transplantation Patients and External Validation on the Medical Information Mart for Intensive Care IV Database: Retrospective Study. J. Med. Internet Res..

[20] Kanas G., Ge W., Quek R.G., Keeven K., Nersesyan K., Arnason J.E. (2022). Epidemiology of diffuse large B-cell lymphoma (DLBCL) and follicular lymphoma (FL) in the United States and Western Europe: Population-level projections for 2020–2025. Leuk. Lymphoma.

[21] Najafabadi M.M., Villanustre F., Khoshgoftaar T.M., Seliya N., Wald R., Muharemagic E. (2015). Deep learning applications and challenges in big data analytics. J. Big Data.

[22] Sekkal R.N., Bereksi-Reguig F., Ruiz-Fernandez D., Dib N., Sekkal S. (2022). Automatic sleep stage classification: From classical machine learning methods to deep learning. Biomed. Signal Process. Control.

[23] Sirocchi C., Bogliolo A., Montagna S. (2024). Medical-informed machine learning: Integrating prior knowledge into medical decision systems. BMC Med. Inform. Decis. Mak..

[24] Müller D., Soto-Rey I., Kramer F. (2022). An analysis on ensemble learning optimized medical image classification with deep convolutional neural networks. IEEE Access.

[25] Anitha J., Akila Agnes S., Pandian S.I.A., Bruntha M., Kumar A., Dubey A.K., Bhatia S., Kumar S.A., Le D.N. (2022). Deep-stacked autoencoder for medical image classification. Evolving Predictive Analytics in Healthcare: New AI Techniques for Real-Time Interventions.

[26] Chen Z., Tong L., Qian B., Yu J., Xiao C. (2021). Self-attention-based conditional variational auto-encoder generative adversarial networks for hyperspectral classification. Remote Sens..

[27] Fu Y., Huang Z., Deng X., Xu L., Liu Y., Zhang M., Liu J., Huang B. (2025). Artificial Intelligence in Lymphoma Histopathology: Systematic Review. J. Med. Internet Res..

[28] Hashimoto N., Ko K., Yokota T., Kohno K., Nakaguro M., Nakamura S., Takeuchi I., Hontani H. (2022). Subtype classification of malignant lymphoma using immunohistochemical staining pattern. Int. J. Comput. Assist. Radiol. Surg..

[29] Ogundokun R.O., Odusami M., Sisodia D.S., Awotunde J.B., Tiwari D.P. (2022). A Novel PCA-Logistic Regression for Intrusion Detection System. Proceedings of the International Conference on Information Systems and Management Science.

[30] Kurita T. (2021). Principal component analysis (PCA). Computer Vision: A Reference Guide.

[31] Ogundokun R.O., Awotunde J.B., Akande H.B., Lee C.C., Imoize A.L. (2024). Deep transfer learning models for mobile-based ocular disorder identification on retinal images. Comput. Mater. Contin..

[32] Cihan P., Saygılı A., Ermutlu C.Ş., Aydın U., Aksoy Ö. (2024). AI-aided cardiovascular disease diagnosis in cattle from retinal images: Machine learning vs. deep learning models. Comput. Electron. Agric..

[33] Volovăț S.R., Boboc D.-I., Ostafe M.-R., Buzea C.G., Agop M., Ochiuz L., Rusu D.I., Vasincu D., Ungureanu M.I., Volovăț C.C. (2025). Utilizing Vision Transformers for Predicting Early Response of Brain Metastasis to Magnetic Resonance Imaging-Guided Stage Gamma Knife Radiosurgery Treatment. Tomography.

[34] Esteva A., Kuprel B., Novoa R.A., Ko J., Swetter S.M., Blau H.M., Thrun S. (2017). Dermatologist-level classification of skin cancer with deep neural networks. Nature.

[35] Dietterich T.G. (2000). Ensemble methods in machine learning. International Workshop on Multiple Classifier Systems.

[36] Ganaie M.A., Hu M., Malik A.K., Tanveer M., Suganthan P.N. (2022). Ensemble deep learning: A review. Eng. Appl. Artif. Intell..

[37] Xie J., Liu R., Luttrell J., Zhang C. (2019). Deep learning based analysis of histopathological images of breast cancer. Front. Genet..

[38] Xie X., Wang X., Liang Y., Yang J., Wu Y., Li L., Sun X., Bing P., He B., Tian G. (2021). Evaluating cancer-related biomarkers based on pathological images: A systematic review. Front. Oncol..

[39] Chen T., Guestrin C. Xgboost: A scalable tree boosting system. Proceedings of the 22nd ACM Sigkdd International Conference on Knowledge Discovery and Data Mining.

[40] Zhu J., Liu M., Li X. (2022). Progress on deep learning in digital pathology of breast cancer: A narrative review. Gland Surg..

[41] Abdel-Nabi H., Ali M., Awajan A., Daoud M., Alazrai R., Suganthan P.N., Ali T. (2023). A comprehensive review of the deep learning-based tumor analysis approaches in histopathological images: Segmentation, classification and multi-learning tasks. Clust. Comput..

[42] Kather J.N., Krisam J., Charoentong P., Luedde T., Herpel E., Weis C.A., Gaiser T., Marx A., Valous N.A., Ferber D. (2019). Predicting survival from colorectal cancer histology slides using deep learning: A retrospective multicenter study. PLoS Med..

[43] Komura D., Ishikawa S. (2018). Machine learning methods for histopathological image analysis. Comput. Struct. Biotechnol. J..

[44] Swerdlow S.H., Campo E., Pileri S.A., Harris N.L., Stein H., Siebert R., Advani R., Ghielmini M., Salles G.A., Zelenetz A.D. (2016). The 2016 revision of the World Health Organization classification of lymphoid neoplasms. Blood.

[45] Campanella G., Hanna M.G., Geneslaw L., Miraflor A., Werneck Krauss Silva V., Busam K.J., Brogi E., Reuter V.E., Klimstra D.S., Fuchs T.J. (2019). Clinical-grade computational pathology using weakly supervised deep learning on whole slide images. Nat. Med..

[46] Bejnordi B.E., Veta M., Van Diest P.J., Van Ginneken B., Karssemeijer N., Litjens G., Van Der Laak J.A., Hermsen M., Manson Q.F., Balkenhol M. (2017). Diagnostic assessment of deep learning algorithms for detection of lymph node metastases in women with breast cancer. JAMA.

[47] Jha N.K., Bhushan B., Aurangzeb K. (2025). Intelligent Green AI Technologies for Promoting Eco-Friendly and Sustainable Smart Cities. Establishing AI-Specific Cloud Computing Infrastructure.

[48] Bandi P., Geessink O., Manson Q., Van Dijk M., Balkenhol M., Hermsen M., Bejnordi B.E., Lee B., Paeng K., Zhong A. (2018). From detection of individual metastases to classification of lymph node status at the patient level: The camelyon17 challenge. IEEE Trans. Med. Imaging.

